# Advanced biliary tract carcinomas: a retrospective multicenter analysis of first and second-line chemotherapy

**DOI:** 10.1186/1471-230X-14-143

**Published:** 2014-08-13

**Authors:** Frédéric Fiteni, Marine Jary, Franck Monnien, Thierry Nguyen, Eric Beohou, Martin Demarchi, Erion Dobi, Francine Fein, Denis Cleau, Serge Fratté, Virginie Nerich, Franck Bonnetain, Xavier Pivot, Christophe Borg, Stefano Kim

**Affiliations:** 1 Department of Medical Oncology, Jean Minjoz University Teaching Hospital, 3 Boulevard Alexander Fleming, Besançon F-25030, France; 2 University Hospital of Besançon, Methodology and quality of life in oncology unit, Besançon, France; 3 INSERM, Unit 1098, University of Franche-Comté, Besançon, Besançon, France; 4 Hospital of Belfort-Montbeliard, Department of Medical Oncology, Montbeliard, France; 5 Department of 344 Gastroenterology, University Hospital of Besançon, Besançon, France; 6 Hospital of Vesoul, Department of Gastroenterology, Vesoul, France; 7 University Hospital of Besançon, Department of Pharmacy, Besançon, France; 8 EA 3181 University of Franche-Comté, Besançon, France; 9 Clinical Investigational Center, CIC-1431, University Hospital of Besançon, Besançon, France

## Abstract

**Background:**

Gemcitabine/Cisplatin (Gem/CDDP) combination has demonstrated a clear survival advantage over gemcitabine alone and has become a new standard in advanced Biliary Tract Carcinoma (aBTC). However, Gemcitabine/Oxaliplatin (GEMOX) combination and Gemcitabine/Carboplatin (Gem/Carb) combination regimens have shown efficacy in phase II trials and there is no comparative study between different platinum salts.

We assessed the efficacy and safety of different platinum-based chemotherapies at first line in aBTC patients. We also analysed the second-line chemotherapy.

**Methods:**

Sixty-four consecutive patients with aBTC diagnosed between 1998 and 2010 were included for analysis. At first line chemotherapy, 44 patients received one day GEMOX regimen (gemcitabine 1000 mg/m^2^ and oxaliplatin 100 mg/m^2^ Day 1, every 2 weeks), and 20 patients received Gem/Carb regimen (gemcitabine at 1000 mg/m^2^ Days 1 and 8 with carboplatin delivered according to an area-under-the-curve (AUC) 5 at day 1, every 3 weeks). At second line, a total of 16 patients received a fluoropyrimidine-based chemotherapy.

**Results:**

With GEMOX regimen, median progression-free survival (PFS) was 3.7 months (95%CI, 2.4 to 5) and median overall survival (OS) was 10.5 months (95%CI, 6.4 to14.7). The main toxicity was peripheral neuropathy (20% grade 2 and 7% grade 3). Grade 3/4 haematological toxicities were rare.

With Gem/Carb regimen, PFS was 2.5 months (95%CI, 2.1 to 3.7) and OS was 4.8 months (95%CI, 3.7 to 5.8). The main grade 3/4 toxicities were haematological: anaemia (45%), thrombocytopenia (45%), and neutropenia (40%).

At second-line, fluoropyrimidine-based chemotherapy was feasible in only a fourth of the patients. The median OS was 5.3 months (95%CI, 4.1 to 6.6), and median PFS was 4.0 months (95%CI, 2.6 to 5.5).

**Conclusions:**

One day GEMOX regimen has a favourable toxicity profile and could be an alternative to standard Gem/CDDP regimen, in particular in unfit patients for CDDP.

At second-line, selective patients may benefit from fluoropyrimidine-based chemotherapy.

## Background

Biliary tract carcinomas (BTCs) are invasive adenocarcinomas that arise from the epithelial cells of the biliary tree, which comprises intrahepatic and extrahepatic bile ducts, and gallbladder. Even though BTCs are considered as rare tumours, they represent about 30% of the total primary liver cancers with an incidence rate close to that of hepatocarcinoma. Approximately 1200 new cases in the United Kingdom and 9000 in the United States are diagnosed per year. Unfortunately, only a minority of patients diagnosed with these aggressive tumours are at an early resectable stage, and disease recurrence rates are high despite curative-intent surgery. Prognosis of patients with advanced BTC is extremely poor with OS less than 1 year [1,2].

Chemotherapy represents a palliative treatment option for patients with advanced disease with significant benefit in OS and quality of life (QoL) over best supportive care (BSC) alone [3].

Different single or combination-drug regimens have demonstrated some activity in BTCs, including fluoropyrimidines, gemcitabine, and platinum salts [3-7]. A pooled analysis from Eckel *et al.* included 104 trials comprising 2810 patients. Even though only 3 small randomized trials were included in this study, they suggested gemcitabine combined with cisplatin or oxaliplatin as the most active, and therefore they were considered as the provisional standard regimens in aBTC [8].

Different GEMOX regimens were assessed in several phase II clinical trials with OS no longer than 12 months [9-12]. In 2010, a randomized multicenter phase III ABC-02 trial established Gem/CDDP as a standard regimen in aBTC. OS was 11.7 months in favour to combination arm compared to 8.1 months in gemcitabine arm (HR, 0.64; 95%CI 95%, 0.52 to 0.80; P < 0.001) [13]. Before ABC-02 trial results, our multidisciplinary committee approved Gem/Carb combination as standard regimen in aBTC up to December 2003, and biweekly GEMOX regimen thereafter, based in existing data at that time.

In second-line, in our knowledge, no trial assessed the benefit of chemotherapy over BSC in aBTCs. Even though there is no clear evidence-based data for a standard regimen in second line, fluoropyrimidine-based chemotherapies are largely applied.

The aim of this retrospective study was to evaluate the OS, PFS, and safety in patients with aBTCs treated in first line chemotherapy by modified one day GEMOX or Gem/Carb regimen in our institution comprising three different hospitals.

We also analysed the efficacy in terms of survival of second line chemotherapy based on fluoropyrimidines.

## Methods

### Study population

Between 1998 and 2010, 64 consecutive patients were retrospectively included in two general hospitals and one university hospital belonging to IRFC-FC (Institut Régional Fédératif du Cancer de Franche-Comté).

Patients were eligible if they had histologically documented BTC including intrahepatic BTC, extrahepatic BTC and gallbladder cancer, unresectable or metastatic disease, and received at least one cycle of chemotherapy.

### Treatment

At first line, patients with aBTC received Gem/Carb regimen until December 2003. From January 2004 to 2010, all patients received GEMOX regimen. Twenty patients received Gem/Carb regimen (gemcitabine at 1000 mg/m^2^ on days 1 and 8, with carboplatin dosed at an AUC of 5 on day 1 of a 21-day cycle), and 44 patients received GEMOX regimen (gemcitabine 1000 mg/m2 on day 1, with oxaliplatin 100 mg/m^2^ on day 1, every 2 weeks).

At second line, 16 of 64 patients reviewed (25%) received chemotherapy; all regimens were based on 5FU/leucovorin (LV5FU2) or capecitabine. Eleven patients received a mono-chemotherapy (either LV5FU2 or capecitabine) and 5 patients a bi-chemotherapy (either FOLFOX or FOLFIRI).

### Statistical analysis

The primary end point was the treatment efficacy of first line chemotherapy in terms of OS. Secondary endpoints were PFS and toxicity of the GEMOX and Gem/Carb regimens as first-line therapy, treatment efficacy of second-line chemotherapy in terms of OS and PFS. OS was defined as the time from the first chemotherapy to death from all causes. PFS was defined as the time from the first chemotherapy to the earliest date of disease progression (local, regional, distant and second cancer), death (from all causes) or data cut-off (from all causes). OS and PFS were estimated using Kaplan Meier method and described by median with 95% Confidence Interval (CI). Survival curves were compared using log-rank test.

Safety was reported for all subjects who received at least one dose of chemotherapy.

The ethical committee, Comité de protection des personnes Est-II, approved the protocol.

## Results

### Patients

Patient’s baseline characteristics are presented in Table 1. The population included 38 males and 26 females (ratio, 1.46), with a mean age of 65 years (range, 34–84). Twenty-nine patients presented intrahepatic BTC, 15 patients presented extrahepatic BTC, and 20 patients had gallbladder cancer. No ampulloma was included in our cohort. Twenty-nine patients were treated with prior tumour resection, and 9 patients received prior radiotherapy. Most patients (74%) were in good performance status at diagnosis (Eastern Cooperative Oncology Group – Performance Status, ECOG-PS 0–1) (Table 1).

**Table 1:** Patient and tumour characteristics

**Characteristics n (%)**	**GEMOX (n = 44)**	**Gem/Carb (n = 20)**	**p**
	**n (%)**	**n (%)**	
*Year of diagnosis*	2004-2010	1998-2003	
*Gender, no (%)*			
Male	29 (66)	9 (45)	0.17
Female	15 (34)	11 (55)	
*Age*			
<=67	19 (43)	12 (60)	0.28
>67	25 (57)	8 (40)	
*ECOG PS, no. (%)*			
0-1	34 (77)	14 (70)	0,75
2-3	10 (23)	6 (30)	
*Primary tumour location*			
Gallbladder	12 (27)	8 (40)	0.59
Intrahepatic bile ducts	21 (48)	8 (40)	
Extrahepatic bile ducts	11 (25)	4 (20)	
Ampulla of Vater	0 (0)	0 (0)	
*Prior treatment for BTC*			
Surgery	22 (50)	7 (37)	0.40
Radiotherapy	5 (11)	4 (21)	
*Number of metastatic sites*			
1	26 (59)	11 (58)	0.81
>1	18 (41)	8 (42)	

*Abbreviations*: *GEMOX* gemcitabine and oxaliplatine combination regimen, *Gem/Carb* gemcitabine and carboplatin combination regimen, *ECOG-PS* Eastern Cooperative Oncology Group – Performance Status, *BTC* biliary tract carcinomas.

### Efficacy

**Figure 1:**
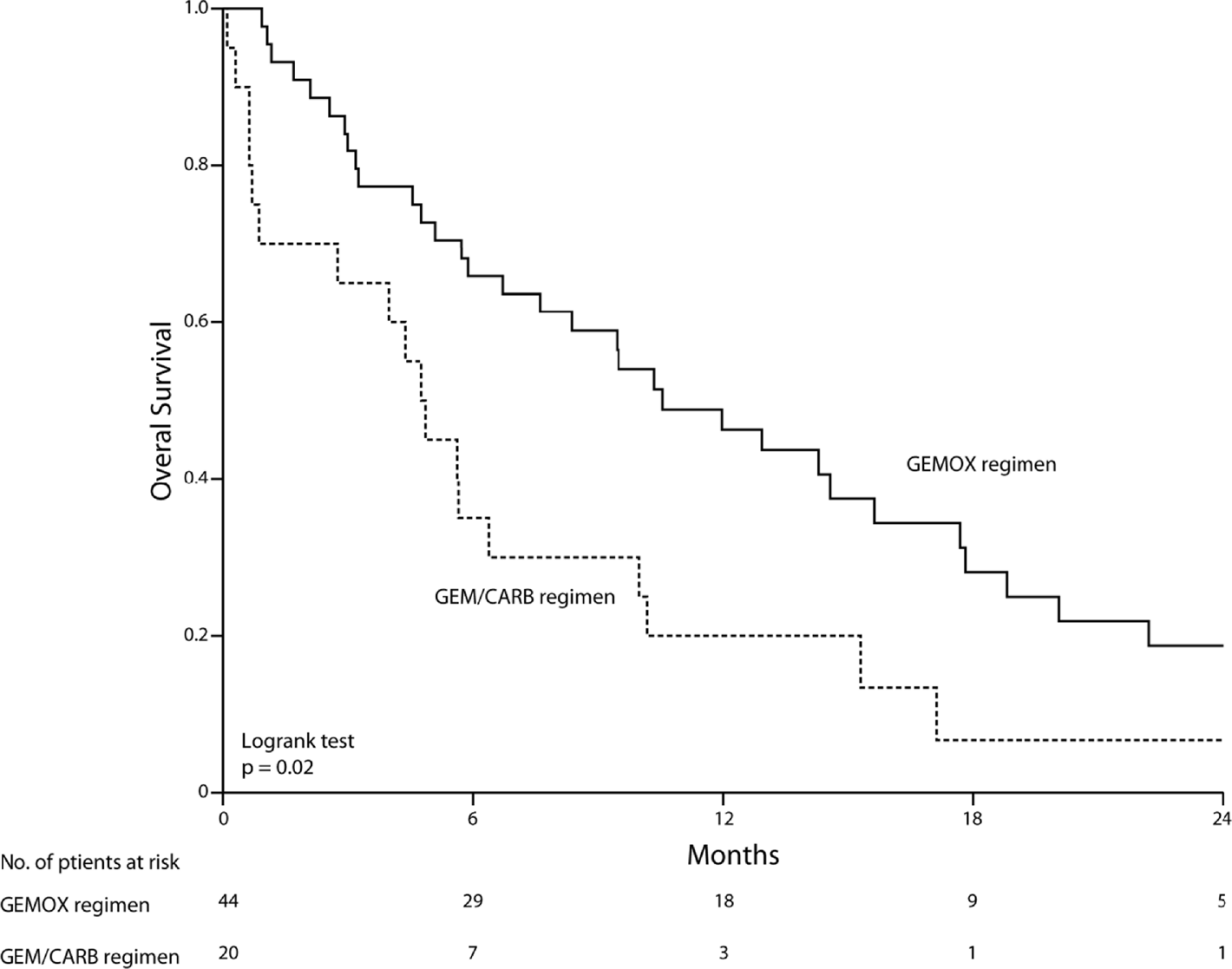
**Overall survival.** GEMOX regimen (n = 44) and Gem/Carb regimen (n = 20).

**Figure 2:**
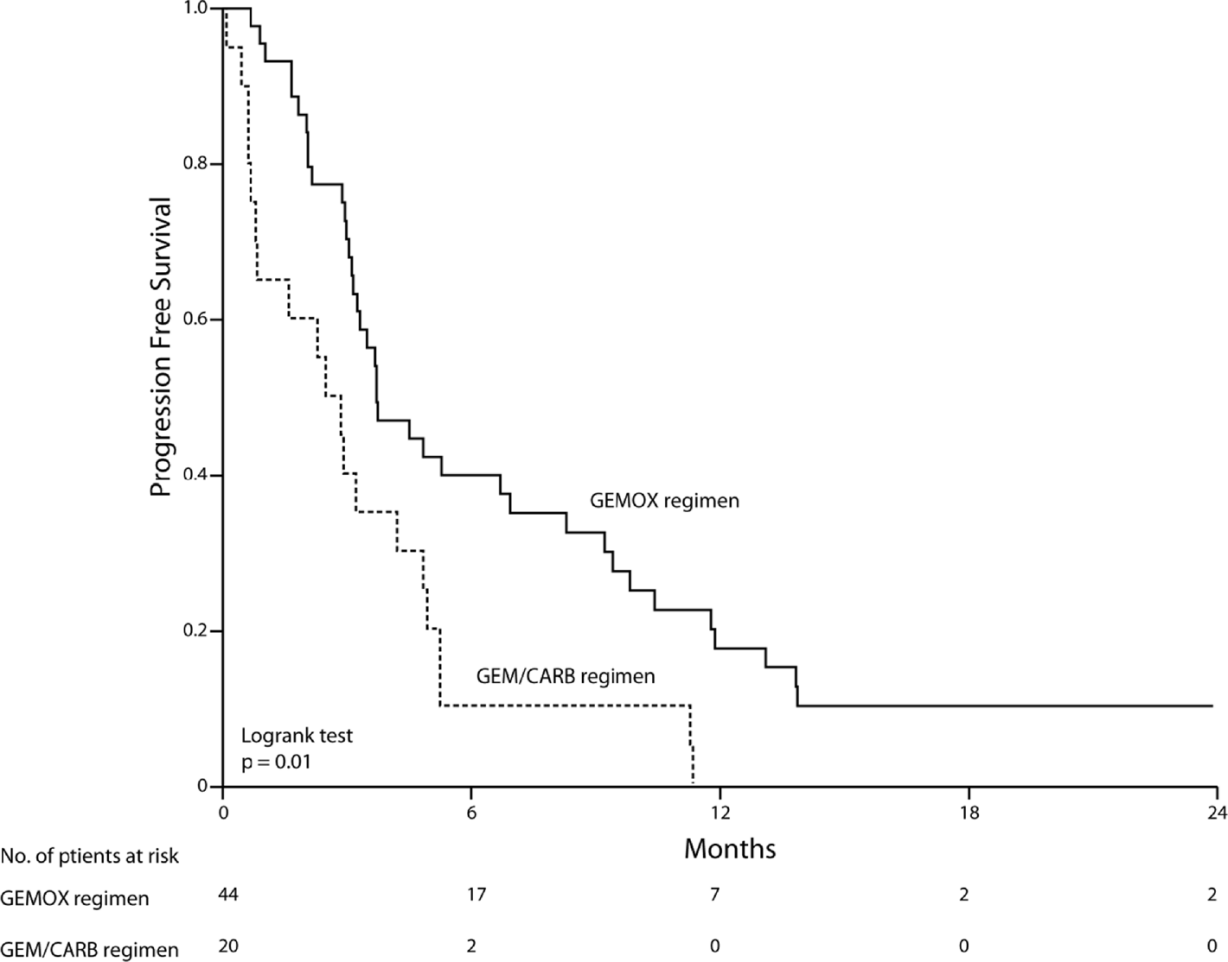
**Progression free survival.** GEMOX regimen (n = 44) and Gem/Carb regimen (n = 20).

Twenty patients received Gem/Carb regimen. Mean age was 62 years (range 34–78) with 9 males and 11 females (ratio, 0.82). Fourteen patients (70%) presented ECOG-PS of 0–1 at diagnosis. All 20 patients were dead at the time of analysis. (Table 1) There were 1 CR (5%), 1 PR (5%), 6 SD (30%) and 12 (60%) progression disease (PD). Median OS was 4.8 months (95%CI, 3.7 to 5.8) (Figure 1), and median PFS was 2.5 months (95%CI, 2.1 to 3.7) (Figure 2). One patient who had primarily unresectable disease underwent curative-intent surgery after chemotherapy. Recurrence free survival (RFS) in this patient was 11.9 months.

Forty-four patients received GEMOX regimen. Mean age was 66 years, (range 47–84 years) with 29 males and 15 females (ratio, 1.93). Thirty-four patients (77%) presented ECOG-PS of 0–1 at diagnosis. Eleven patients were alive at the time of analysis with a median follow-up of 35 months. (Table 1) There were 3 CR (7%), 5 PR (11%), 9 SD (20%) and 27 PD (61%). Median OS was 10.5 months (95%CI, 6.4 to 14.7) (Figure 1), and median PFS was 3.7 months (95%CI, 2.4 to 5) (Figure 2). In 2 patients the tumour became resectable after chemotherapy. Their RFS was 13.3 and 14.3 months.

Among 64 patients treated with platinum/gemcitabine combinations as front-line regimen, 8 patients (13%) had a median OS > 24 months.

### Safety

Chemotherapy was administered until progression, unacceptable toxicity, or curative-intent surgery. Two patients in GEMOX cohort stopped the chemotherapy after 37th cycle due to a long lasting complete response of metastases. The median relative dose intensities in GEMOX cohort were 96% and 93% for gemcitabine and oxaliplatine, respectively; and in the Gem/Carb cohort were 97% and 95% for gemcitabine and carboplatin, respectively. The main reason for treatment discontinuation was disease progression. Only 3 patients (7%) in GEMOX cohort and one patient (5%) in Gem/Carb cohort stopped the treatment for toxicity. The median number of treatment cycles administered was 7 (range, 1–37) in the GEMOX cohort and 3 (range, 1–7) in the Gem/Carb cohort. More patients in the GEMOX cohort received 16 or more weeks of treatment (49% v 29%).

In GEMOX regimen, the main toxicity was the peripheral neuropathy, present in 59% of patients including 7% with grade 3 neuropathy. Anaemia was observed in 43% (2% grade 3, no grade 4), and thrombocytopenia was observed in 36% (2% grade 3, no grade 4).

Concerning Gem/Carb regimen, the main toxicity was haematological. Anaemia was observed in 75% of patients (40% grade 3, 5% grade 4), thrombocytopenia in 60% (30% grade 3, 15% grade 4), and neutropenia in 50% (30% grade 3, 10% grade 4) (Table 2).

**Table 2:** Adverse events

**Adverse events. No. (%)**	**All grades (1–4)**		**Grade 3**		**Grade 4**	
	**GEMOX**	**Gem/Carb**	**GEMOX**	**Gem/Carb**	**GEMOX**	**Gem/Carb**
	**(n = 44)**	**(n = 20)**	**(n = 44)**	**(n = 20)**	**(n = 44)**	**(n = 20)**
*Haematological*						
Anaemia	19 (43)	15 (75)	1 (2)	8 (40)	0	1 (5)
Thrombocytopenia	16 (36)	12 (60)	1 (2)	6 (30)	0	3 (15)
Neutropenia	4 (9)	10 (50)	0	6 (30)	1 (2)	2 (10)
Febrile neutropenia	1 (2)	0	0	0	0	0
*Non haematological*						
Fatigue	14 (32)	4 (20)	0	2 (10)	0	0
Nausea	10 (23)	3 (15)	0	2 (10)	0	0
Vomiting	6 (14)	4 (20)	0	3 (15)	0	0
Liver toxicity	3 (7)	2 (10)	0	0	0	0
Peripheral neuropathy	26 (59)	0	3 (7)	0	0	0
Infection without neutropenia	9 (21)	1 (5)	0	1 (5)	0	0
Renal toxicity	2 (5)	0	0	0	0	0

*Abbreviations*: *GEMOX* gemcitabine and oxaliplatine combination regimen, *Gem/Carb* gemcitabine and carboplatin combination regimen.

### Second line chemotherapy

Sixteen patients received second line chemotherapy. Median OS was 5.3 months (95%CI, 4.1 to 6.6), and median PFS was 4.0 months (95%CI, 2.6 to 5.5). Median OS was 5.2 months (95%CI, 3.8 to 6.6) for the mono-chemotherapy group as compared to 6.1 months (95%CI, 2.0 to 10.3) in the bi-chemotherapy group. Median PFS was 3.8 months (95%CI, 2.7 to 4.9) for the mono-chemotherapy group as compared to 4.4 months (95%CI, 2.9 to 6.0) in the bi-chemotherapy group. No significant difference was found between the two groups (p = 0.98 and p = 0.68, respectively).

## Discussion

A phase III ABC-02 trial including 410 patients demonstrated overall survival superiority of Gem/CDDP combination over gemcitabine alone, establishing a new standard in front-line chemotherapy for aBTCs (11.7 *vs.* 8.2 months, HR 0.64; 95%CI, 0.52 to 0.80; P < 0.001). The PFS was 8.0 months in the Gem/CDDP arm versus 5.0 months in the control arm (P < 0.001). Adverse events were similar in both groups, with the exception of more neutropenia in the combination arm [13].

Oxaliplatin is active as monotherapy in patients with BTC [7]. Synergic antitumoral effect with gemcitabine was seen in preclinical studies [14]. Several phase II trials support the use of oxaliplatin combined to gemcitabine in aBTC [9-12]. André *et al.* conducted a multinational phase II trial and 70 patients were treated by GEMOX regimen (gemcitabine 1000 mg/m^2^ Day 1, and oxaliplatin 100 mg/m^2^ Day 2, every two weeks). Median OS rate was 8.8 months (95%CI, 6.9 to 11.1) and tolerance profile was favourable. Sharma *et al.* evaluated efficacy of modified gemcitabine and oxaliplatin (mGEMOX) over BSC or fluorouracil and folinic acid (FUFA) (gemcitabine 900 mg/m2 and oxaliplatin 80 mg/m2, both Day 1, and 8, every 3 weeks). Median OS was 9.5, 4.5, and 4.6, months for mGEMOX, BSC, and FUFA (P = 0.039), respectively [15]. A pooled analysis of 104 trials with 2810 patients suggests that gemcitabine and platinum combination, including oxaliplatin may improve survival compared to other regimens [8]. Superiority of one platinum salt over another in this setting was not demonstrated, and there is no clinical trial with direct comparison between different platinum salts in BTCs, nether between different GEMOX regimens.

In our study, we used biweekly GEMOX regimen. However it is slightly different to André’s one. Both, gemcitabine and oxaliplatin are administered at day 1 with gemcitabine preceding oxaliplatin. Our result, based in non-selected patients, showed an OS of 10.5 months. Even though our cohort has several limitations like a small number of patients, retrospective analysis, and no control arm, this OS result is still encouraging since there were more ECOG-PS 2 patients and a higher median age than in randomized or well designed phase II trials. The treatment was well tolerated. The main toxicity was peripheral neuropathy observed in 59%. Oxaliplatin was stopped in 3 patients for grade 3 peripheral neuropathy. Other grade 3 toxicities were rare and no grade 4 was reported. In ABC-02 study, more than 70% of patients presented grade 3/4 toxicities in Gem/CDDP arm, and significantly more haematological toxicities were observed compared to gemcitabine alone.

Gem/Carb combination was successfully assessed in several phase III trials for tumours from different sites (e.g., lung, and bladder cancers) [16-18]. In aBTCs, Williams and al conducted a phase II trial (gemcitabine 1000 mg/m^2^ and 8 and carboplatin AUC 5 Day 1, every three weeks) and 48 patients were consecutively included with a median OS of 10.6 months (95%CI, 8.8 to14.2) [19]. Our cohort did worse with the same treatment schedule. Even though 20 patients are certainly underpowered to estimate OS, it was only 4.8 months (95%CI, 3.7 to 5.8). Grade 3/4 toxicities were frequent, including grade 4 haematological toxicities.

Health related QoL is a major concern in this palliative setting. Number of visits is in favour of our GEMOX regimen with 2 visits per month compared to 3 in the same period for Gem/CDDP and Gem/Carb regimens, and 4 visits for classic GEMOX regimens. However, the benefit of a limited number of visits in terms of QoL was not demonstrated. Moreover, compared to standard Gem/CDDP, GEMOX regimen needs no hyperhydration and has probably minor risks of renal complication. GEMOX regimen is already considered as a standard arm in new studies like BINGO trial, which compares GEMOX plus cetuximab versus GEMOX alone [20].

In second line, there are limited clinical data to suggest a clinical benefit of chemotherapy in aBTC and there is no regimen considered as standard in this setting. However, anticancer activity of fluoropyrimidines in BTC is well known [6]. Recently, a phase II trial evaluated capecitabine and oxaliplatin combination as second-line regimen in pancreatic cancers and BTCs. All patients progressed after gemcitabine-based regimen. Forty patients were assessed including 17 BTCs. DCR was 22%, PFS was 15 weeks (95%CI, 6.6 to 23.3), and OS was 19 weeks (95%CI, 10.4 to 27.5) [21]. These results are similar to that observed in our cohort with a PFS of 4 months (95%CI, 2.6 to 5.5) and OS of 5.3 months (95%CI, 4.1 to 6.6). Even though only a fourth of patients was able to receive second-line chemotherapy, fluoropyrimidine-based chemotherapy may add clinical benefit in this selected population since median survival rates were not negligible. However, the clinical interest on the administration of a more toxic bichemotherapy over monochemotherapy in this palliative setting needs to be demonstrated.

## Conclusion

In conclusion, one day GEMOX regimen has a favourable toxicity profile and could be an alternative to standard Gem/CDDP regimen, in particular in unfit patients for CDDP.

At second-line, selective patients may benefit from fluoropyrimidine-based chemotherapy.

## Competing interests

Financial competing interests: the authors declare no financial competing interests.

Non-financial competing interests: the authors declare no non-financial competing interests.

## Authors’ contributions

FF conceived the study, participated in its design, acquisition of data, statistical analysis, and helped to draft the manuscript. TN conceived the study, participated in study design, acquisition of data, and helped to draft the manuscript. FM participated in statistical analysis and helped to draft manuscript. EB participated in acquisition of data and statistical analysis. MJ participated in acquisition of data and helped to draft the manuscript. MD participated in the interpretation of data and helped to draft the manuscript. ED participated in acquisition of data. FFein participated in acquisition of data. DC participated in acquisition of data. SF participated in acquisition of data. VN participated in the interpretation of data and statistical analysis. FB participated in the interpretation of data, statistical analysis, and helped to draft the manuscript. XP participated in the interpretation of data and helped to draft the manuscript. CB conceived the study, participated in its design, interpretation of data, and helped to draft the manuscript. SK conceived the study, participated in its design, interpretation of data, coordination, and helped to draft the manuscript. All authors read and approved the final manuscript.

## Pre-publication history

The pre-publication history for this paper can be accessed here:

http://www.biomedcentral.com/1471-230X/14/143/prepub
